# Transcranial Alternating Current Stimulation Enhances Individual Alpha Activity in Human EEG

**DOI:** 10.1371/journal.pone.0013766

**Published:** 2010-11-01

**Authors:** Tino Zaehle, Stefan Rach, Christoph S. Herrmann

**Affiliations:** 1 Department of Neurology, Otto-von-Guericke University, Magdeburg, Germany; 2 German Center for Neurodegenerative Diseases (DZNE), Magdeburg, Germany; 3 Experimental Psychology Lab, Carl von Ossietzky Universität, Oldenburg, Germany; University of Groningen, Netherlands

## Abstract

Non-invasive electrical stimulation of the human cortex by means of transcranial direct current stimulation (tDCS) has been instrumental in a number of important discoveries in the field of human cortical function and has become a well-established method for evaluating brain function in healthy human participants. Recently, transcranial *alternating* current stimulation (tACS) has been introduced to directly modulate the ongoing rhythmic brain activity by the application of oscillatory currents on the human scalp. Until now the efficiency of tACS in modulating rhythmic brain activity has been indicated only by inference from perceptual and behavioural consequences of electrical stimulation. No direct electrophysiological evidence of tACS has been reported. We delivered tACS over the occipital cortex of 10 healthy participants to entrain the neuronal oscillatory activity in their individual alpha frequency range and compared results with those from a separate group of participants receiving sham stimulation. The tACS but not the sham stimulation elevated the endogenous alpha power in parieto-central electrodes of the electroencephalogram. Additionally, in a network of spiking neurons, we simulated how tACS can be affected even after the end of stimulation. The results show that spike-timing-dependent plasticity (STDP) selectively modulates synapses depending on the resonance frequencies of the neural circuits that they belong to. Thus, tACS influences STDP which in turn results in aftereffects upon neural activity.

The present findings are the first direct electrophysiological evidence of an interaction of tACS and ongoing oscillatory activity in the human cortex. The data demonstrate the ability of tACS to specifically modulate oscillatory brain activity and show its potential both at fostering knowledge on the functional significance of brain oscillations and for therapeutic application.

## Introduction

The brain's ability to generate and sense temporal information is a prerequisite for perception, action, and cognition [1]. This temporal information is embedded in oscillations that exist at many different time scales [2]. In the healthy awake human at rest with eyes closed (“relaxed wakefulness”), the most prominent component in the EEG is the 8 to 12 Hz alpha rhythm, known since the pioneering work of Berger [3]. The occipital alpha rhythm has been linked to cognition [4] and working memory [5] and is the main target of training-induced alterations by operant conditioning in the context of biofeedback [6]. Alpha power increases from early childhood to adulthood and decreases beyond the age of 50-60 years, a decline that has been related to age related neurological disorders and not to age per se. Furthermore, children with poorer education, reading/writing disabilities, spelling disabilities, and neurological disorders show significantly less alpha power [7], and neural modulation of alpha power is strongly affected in patients with Alzheimer's disease [8].

The causal nature of a close relationship between EEG alpha oscillations and human behavior has been pointed out by demonstrating that artificially enhanced alpha power by repetitive trancranial magnetic stimulation (rTMS) [9], [10] or neurofeedback training [11], [12] can improve cognitive task performance. Furthermore, endogenous occipital alpha power preceding a visual stimulus determines the perceptual fate of the stimulus [13]–[15]. Thus, ongoing oscillatory alpha activity even in the absence of stimulus input or motor output can be seen as an index of internal states of the brain and has predictive power for subsequent sensory experience or cognitive processing [16], [17]. Accordingly, it would be desirable to modulate human alpha activity in cases of cognitive disabilities that arise from pathologically low or high brain oscillations in this frequency range.

Transcranial electrical stimulation of the human cortex has proven to be a useful method in neuroscience [18], [19]. Transcranially applied *direct* current stimulation (tDCS) causes polarization and depolarization of the neuronal areas under the anode and cathode, respectively – thus modulating excitability of the cortex [20]–[24]. The application of trancranial *alternating* current stimulation (tACS) is potentially capable of interacting with rhythmic neuronal activity and has perceptual [25] and behavioural [26] consequences. By application of tACS over the occipital cortex, Kanai and colleagues were able to induce phosphene perception in a frequency dependent manner [25] (but see [27] for a controversial discussion). In the same vein, Pogosyan and co-workers related tACS over the motor cortex to stimulation-specific alterations in voluntary movement [26]. These effects strongly indicated an interaction of tACS and frequency specific underlying endogenous oscillations.

**Figure 1:**
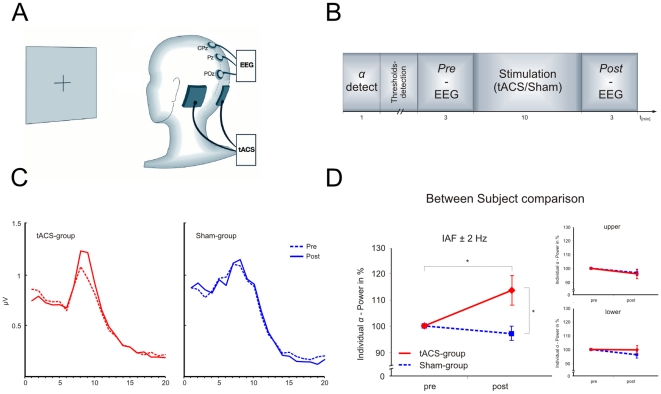
Stimulation details and results. A: Location of stimulation and EEG electrodes: The tACS electrodes were placed bilaterally over the occipital cortex (PO9, PO10, international 10/10 system); EEG is measured from parieto-occipital midline electrodes CPz, Pz, and POz. *B: Timeline of experimental events:* The experiment started with the determination of the individual alpha frequency during a 1-minute period in which the participants were in a relaxed state with eyes closed followed by an evaluation of an individual phosphene threshold. Subsequently, the participants performed a simple detection task for 16 minutes. During this period, EEG recording was stopped after 3 minutes (*Pre*-measure) and a 10 minute stimulation (either tACS or Sham) was given, followed by a further 3 minute EEG recording (*Post*-measure). *C: Group averaged EEG activity:* Average FFT power spectra for the 3 minute intervals preceding (*pre*, dotted line) and following (*post*, solid line) the stimulation condition separately for the tACS-group (left) and the Sham-group (right). *D: mean individual alpha amplitude for tACS and sham group:* There is an increase in individual alpha power from pre- to post-stimulus measurement in the subjects that received tACS (solid line), but not for the subjects that received sham-stimulation (dotted line). No tACS related alpha modulation can be observed in the upper and lower frequency band. Data are normalized to the pre-stimulation alpha power. Asterisks indicate statistical significance. Data are the means ±s.e.m.

The aim of the present study was to provide direct electrophysiological evidence for the interaction of tACS and endogenous oscillatory neural activity. We applied tACS over the occipital cortex at the individual alpha frequency of 10 healthy participants, as well as a sham stimulation in a separate sample of 10 participants, and measured the alpha power from 3 parieto-central midline electrodes (Figure 1a). We hypothesized that tACS would interact with the ongoing neuronal activity and entrain the individual alpha oscillations.

## Materials and Methods

### Participants

Twenty healthy subjects (10 female) with a mean age of 25.85±4.5 years, and free of medication, participated in the study after having given written informed consent. Participants were divided in an alternating odd-even fashion into two groups, one experimental (EG) and one control group (CG). Groups did not statistically differ in age (EG: 27.3±4.8 years; CG: 24.4±3.9 years; non-directional independent t-statistic: *t*_18_ = 1.48, *P* = 0.16), gender (EG: 4 female; CG: 6 female, *χ* ^2^_1_ = 0.8 (n = 20), *P* = 0.37), or individual alpha frequency (IAF) in their endogenous EEG (EG: 10.41±0.87 Hz, CG: 10.22±0.80 Hz; non-directional independent t-statistic: *t*_18_ = 0.51, *P* = 0.62) . We applied a single blind study. Until the end of the experiment, the participants were not aware whether they received tACS or sham stimulation. The experimental protocol was approved by the local ethics committee.

### EEG

The experiment was performed in an electrically shielded, sound-attenuated, and dimly lit cabin (IAC, Niederkruchten, Germany). For visual stimulation, a TFT monitor was placed outside the cabin behind an electrically shielded window. All devices inside the cabin were battery-operated to avoid line frequency interference.

The electroencephalogram (EEG) was measured from the 3 scalp locations CPz, Pz, and POz, according to the 10–20 System, and amplified using a BrainAmp amplifier (Brain Products, Munich, Germany). An electrode placed on the nose served as reference. Activity was recorded using sintered Ag/AgCl electrodes mounted in an elastic cap (Easycap, Falk Minow, Munich, Germany). Electrode impedances were kept below 5 kΩ;. EEG data were acquired at a sampling rate of 500 Hz and were filtered on-line with a band-pass filter of 0.016–200 Hz with an infinite impulse response (IIR) filter with an attenuation of 12 dB/octave. A fiber-optic cable transferred the digitized EEG to a computer outside the recording cabin. After data storage, an additional finite impulse response (FIR) high-pass filter with a cut-off frequency of 0.5 Hz (60 dB attenuation of direct current (DC) signals) was applied off-line in order to reduce slow shifts in the baseline.

### Electrical stimulation

In the experimental group, trancranial electrical stimulation was applied via two sponge electrodes (5×7 cm) (Neuroconn, Ilmenau, Germany) attached to the head underneath an EEG Recording Cap (EASYCAP, Herrsching, Germany) and placed bilaterally at parieto-occipital locations (PO9 and PO10). The impedance was kept below 10 kΩ. We applied oscillating currents at the IAF of each participant using a battery-operated stimulator system (Eldith, Neuroconn, Ilmenau, Germany).

Generally, the dominant frequency in the scalp EEG of human adults is in the alpha frequency range (8–12 Hz) and can be detected as prominent peak in the frequency spectrum. The alpha frequency varies to a large extent as a function of age, neurological disease, memory performance, brain volume and task demands [28], as well as the genetic make up [29]. Therefore, we used the individual EEG alpha frequency, rather then a fixed frequency range, to determine the stimulation frequency and to measure the tACS-induced cortical modulation. In the control group, sham stimulation was applied. All parameters were the same as in the experimental group except that the stimulator remained off during the stimulation period. All participants underwent a tACS- measure prior to the stimulation experiment to determine the thresholds for phosphenes (visual flashes) and skin sensations induced by tACS. The subsequent tACS stimulation in the experimental group was set below these thresholds. This excludes the possibility that participants were able to determine whether they were in the sham or in the stimulation group. A debriefing after the experiment was carried out in order to find out whether stimulation was felt by the participants.

### Design

The procedure is illustrated in Figure 1b. After application of EEG and tACS electrodes, the experiments started with the evaluation of the individual alpha peak frequency. For this purpose the participants were asked to relax and close their eyes while the spontaneous EEG was recorded for 1 minute. Subsequently, the EEG signal was analysed (Vision Analyzer, Brain Products GmbH, München). To this end, the raw EEG was split into 1 second segments. For each segment, a fast Fourier transformation (FFT) was performed and the resulting 60 spectra were averaged. The prominent alpha peak was visually detected and its frequency used for the following procedure.

In the next step, we defined the tACS-induced thresholds for skin sensation and phosphene perception for each participant. We avoided phosphenes to rule out potential retinal contributions to the effects of cortical modulation [30], [31]. For that purpose, we applied tACS stimulation at the individual alpha frequency for 1 second at a time and increased the amplitude stepwise by 250 µA starting with 1000 µA and reaching a maximum of 3000 µA. Participants were asked to keep their eyes open and indicate the presence of a sensation. For the remaining experiment, stimulation intensity (1120±489 µA) was kept 250 µA below the lower threshold for either phosphenes or skin sensations. In two participants, the initial stimulation intensity of 1000 µA induced skin sensations. In these cases, the intensity was decreased stepwise by 100 µA until no sensation was elicited. The resulting stimulation intensity (800 µA and 900 µA respectively) was 100 µA below the individual threshold.

Following these pre-measurements, participants performed a visual change detection task for 16 minutes. Participants were instructed to observe a centered cross (diameter 6 deg) on the screen and to indicate a 45° rotation of the cross by pressing a button with the index finger of the dominant hand. The cross rotated after a variable interval ranging in duration from 35 to 45 seconds and remained rotated for 200 ms. Then it rotated back. The task was used to assure a constant level of vigilance.

During the first 3 minutes of the task, the EEG was recorded (*pre-stimulation measure*). During the subsequent 10 minutes, participants received either tACS or sham stimulation, again followed by 3 minutes of EEG recording (*post-stimulation measure*).

### Data analysis

Data analysis was performed using MATLAB 7 (The MathWorks Inc, Natick, MA, USA). For each of the two data sets from each participant (*Pre, Post*) the raw EEG was split into segments of 1 second duration. All segments that contained a visual stimulation change (rotating cross), or a motor response were excluded from further analysis. The first 150 remaining segments of each data set were used. The mean value of each segment was subtracted before further processing in order to avoid DC distortion of the FFT spectra at 0 Hz. Subsequently, absolute spectra were computed via a fast Fourier transform (FFT) for each segment. The resulting 150 spectra were averaged. In order to evaluate tACS-induced cortical modulation, the mean spectral amplitude within the frequency range of the individual alpha frequency (IAF) ±2 Hz was calculated (*alpha band*) and entered into a repeated-measures analysis of variance (ANOVA) with the between subject factor *Group* (tACS-group/Sham-group) and the within subject factor *Measurement* (Pre/Post). Subsequently, post-hoc t-statistics were performed. Additionally, we also analysed surrounding frequency bands of IAF -5 Hz to IAF -3 Hz (*lower band*) and IAF +3 Hz to IAF +5 Hz (*upper band*). For illustration IAF data were subsequently normalized to the alpha power of the pre-stimulation measurement.

## Results

The debriefing after the experiment revealed that stimulation was not felt by any of the participants. Analysis of FFT power spectra demonstrated an elevation of the individual alpha power specifically related to tACS (cf. Figure 1c). A mixed ANOVA with the between subject factor G*roup* (tACS-group/Sham-group) and the within subject factor *Measurement* (Pre/Post) revealed a significant *Group* x *Measurement* interaction (*F*_1,18_ = 8.21, *P* = 0.01). The individual alpha power increased from pre- to post-stimulation measurement in the subjects that received tACS, but not in the subjects that received sham stimulation (cf. Figure 1d).

Post-hoc t-tests showed a significant difference between pre- and post-stimulation measurements in the tACS-Group (*t*_ 9_ = −2.74, *P* = 0.023), but not in the Sham-Group (*t*_ 9_ =  0.9, *P* = 0.39). From pre- to post-stimulation, the averaged individual alpha power was elevated by 14% in the tACS group.

A t-test for independent samples showed a significant difference between the post-measurements of the tACS- and the Sham-Group (*t*_ 18_ =  1.99, *P* = 0.03, one-tailed).

No significant interaction was found in the *lower* (*F*_1,18_ = 0.43, *P* = 0.5), or in the *upper* frequency band (*F*_1,18_ = 0.01, *P* = 0.9), demonstrating that the entrainment of the endogenous neural oscillations was restricted to the specific tACS frequency.

## Discussion

The human brain is a complex dynamic system generating a multitude of oscillatory waves [32]. These neural oscillations are ubiquitous in cortical systems and play an essential role in human cognition [33]. Depending on the specific frequency band, neural oscillations can be linked to a variety of neural processes, including input selection, plasticity, binding, and consolidation [32], as well as cognitive functions including salience detection, emotional regulation, attention and memory [34]. Also the speed of cognitive functions seems to be influence by alpha oscillations [28].

As demonstrated in the present study, endogenous alpha band oscillations can be entrainment of by means of non-invasive tACS. Such entrainment of alpha oscillations might be used to specifically modulate the internal brain state, and hence modulate subsequent perceptual or cognitive performance. Applications of tACS may even be used in therapy in the future. So far, only tDCS has been applied as a potential therapeutic tool for treatment of several neurological and psychiatric disorders [35]–[43], and particularly for the treatment of memory deficits in stroke patients [44], patients with Parkinson's disease [45], and patients suffering from Alzheimer's disease [36], [46]. Given the fact that certain dysfunctions of cognitive processes can result from altered or disrupted neuronal oscillations (see e.g. [47] for a review), the potential therapeutic applications of tACS span a wide range of diseases. Principally, tACS could be applied to patients in analogy to the well established protocols used with tDCS and is therefore easy and save to operate.

Especially for alpha oscillations, recent evidence for a functional role of stable alpha oscillations in memory has been reported. Here, aberrant temporo-parietal alpha activity in early stage Alzheimer's Disease (AD) has been observed, suggesting that amplitude modulation of neuronal oscillation may be important for memory and strongly affected by AD [11]. The possibility to specifically up-regulate neural oscillations could thus be used to systematically alter cognitive and memory performance and therefore to potentially reduce age- and disease-related performance deterioration. However, the possible therapeutic application of tACS need not be limited to alpha activity. Analogous aberrant neural oscillations have been observed in major depressive disorders for the theta band [48], in epilepsy for the beta band [49], [50], and in schizophrenia for the gamma band [51].

Besides its great potential as a tool for therapeutic application, the ability to specifically modulate individual frequency components of endogenous neural oscillations will provide a powerful new method of investigating the functional significance of several oscillatory bands in human cognition. The current findings provide the first evidence for a direct modulation of endogenous neural oscillations by transcranial alternating current stimulation of the human cortex. Until now, the modulatory effect of tACS on endogenous neural oscillations has been indicated only by inference from its perceptual [2], and behavioural [3] consequences. However, two former studies did report electrophysiological consequences of transcranially applied electric stimulation in humans [52], [53]. These authors used tACS at low frequencies combined with tDCS, i.e. added the alternating current onto a direct current (also called anodal trancranial slow oscillation stimulation, tSOS) to improve memory consolidation. However, the polarizing and depolarizing effects of tDCS on the underlying neural tissue [26] in these investigations cannot be disentangled from the specific oscillatory driving of tACS. Therefore, the present study is the first to characterize the ‘pure’ entraining effect of tACS on brain rhythmicity.

**Figure 2:**
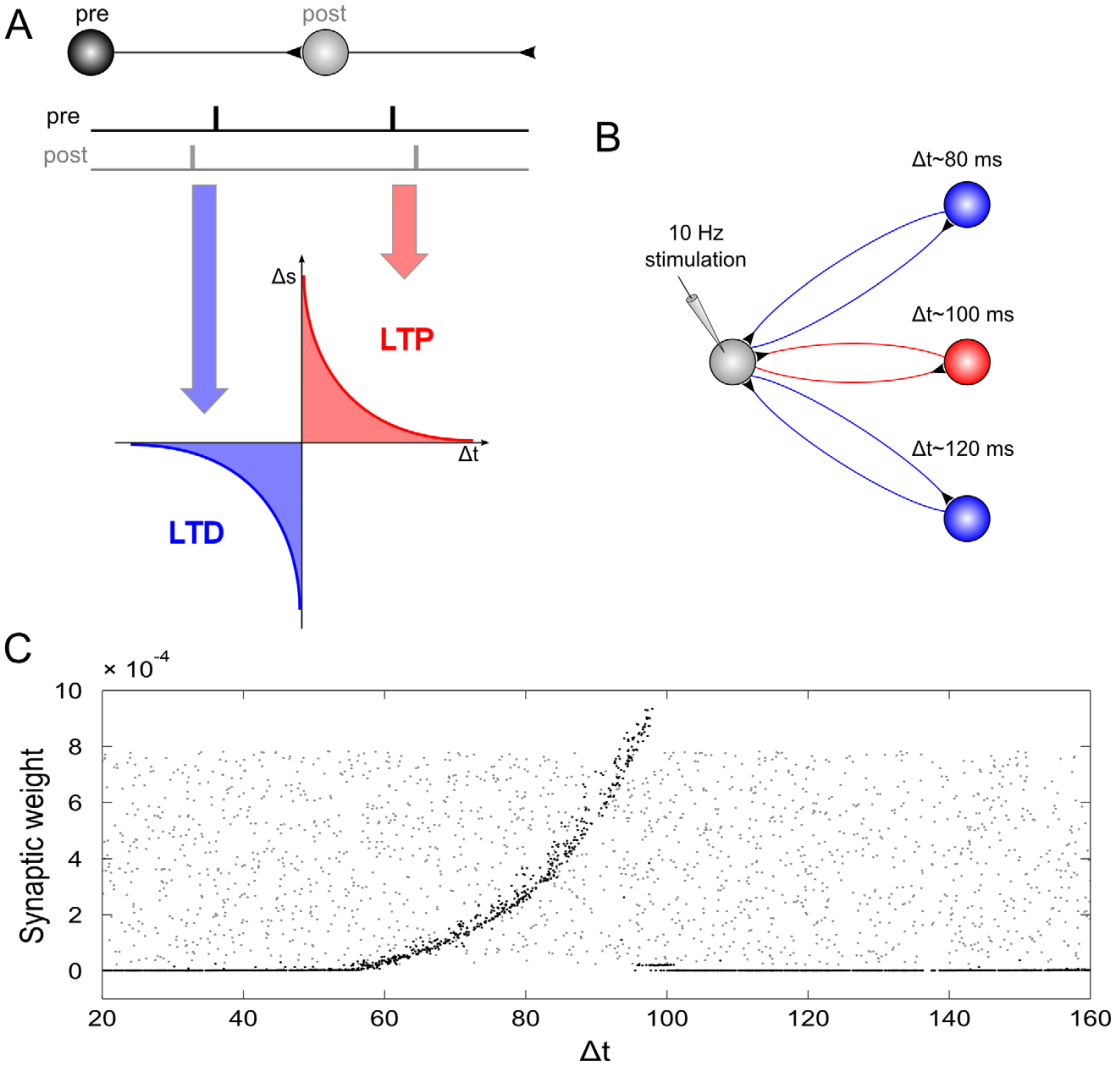
Network simulation of tACS. A: Spike timing dependent plasticity: synaptic weights are increased if a post-synaptic potential follows a pre-synaptic spike (long-term potentiation, LTP) and decreased if a post-synaptic potential occurs prior to a pre-synaptic spike (long-term depression, LTD). *B: Schematic illustration of the network*: A driving neuron establishes a recurrent loop with each neuron of a hidden layer. The total synaptic delay, Δt, (i.e., the sum of both delays of the loop) varied between 20 and 160 ms. The driving neuron was stimulated with a spike train of 10 Hz repetition rate. *C: Synaptic weights of the back-projection as a function of the total synaptic delay of the recurrent loops:* Grey dots display synaptic weights at the start of the simulation, black dots represent synaptic weights after the end of simulation. External stimulation of the driving neuron at 10 Hz resulted in increased weights for recurrent loops with a total delay between 60 and 100 ms, and dramatically reduced synaptic weights for loops with total delays outside this interval. Note, that the highest synaptic weights are observed at 100 ms, i.e., for loops with a resonance frequency near the stimulation frequency.

1tACS that lead to the observed effect of a tACS induced increases in the individuals' EEG alpha amplitude. The first question addresses the issue why tACS works at all, since tDCS at 1mA intensity probably operates below the threshold of cortical neurons, i.e. the stimulation does not directly result in action potentials. Francis et al. (2003) have demonstrated that weak electric fields need to exceed intensities of about 150 µV/mm in order to result in action potentials in hippocampal slices of the rat [54]. Miranda et al. (2006) used inverse modelling to show that 1mA of tDCs leads to about 110 µV/mm of electric fields in cortex, thus probably staying below the threshold for action potentials [55].(Note, however, that already tDCS intensities of 2mA can exceed this threshold.) Even if 1mA of stimulation intensity must be considered sub-threshold, the increased membrane potential of the neurons makes it more likely that the stimulated neurons fire when receiving input from other neurons. The same mechanism must be assumed for tACS which forces the membrane potential to oscillate away from its resting potential towards slightly more depolarized and slightly more hyperpolarized states. During phases of depolarization, tACS can then increase the likeliness of a neuron to fire in response to other neurons. This mechanism has been called stochastic resonance [56] and it seems plausible to assume that this is the cause for observed behavioral effects like phosphenes [2], [57]. The second question concerns how tACS results in changes of neural activity that persist even after stimulation has been turned off. Both, tDCS and repetitive trancranial magnetic stimulation (TMS) also lead to aftereffects of up to one hour duration or longer [58], [59]. For tACS, we believe that this effect originates from synaptic plasticity. Synapses are either strengthened or weakened depending on the exact timing of their input and output activity. When a pre-synaptically arriving action potential precedes a post-synaptic potential, the synapse is strengthened – long-term-potentiation (LTP) of the synapse occurs. If, however, the post-synaptic potential precedes a pre-synaptic potential, the latter cannot have been causal for the former and the synapse is weakened – long-term-depression (LTD) occurs. These mechanisms have been referred to as spike-timing-dependent plasticity (STDP) [60]. Within a neural network, multiple pathways exist that lead from one neuron via other neurons back to the same neuron. These neural circuits can be considered neural oscillators, since it takes a certain amount of time for spikes to run around these circuits. If repetitive input reaches such circuits, the strength of their neural response depends upon the frequency at which they are stimulated, a phenomenon known as neural resonance [61]. The resonance frequency of such circuits is reciprocal to the time that spikes need to run around the circuit. According to the STDP rule, synapses of those circuits that have a resonance frequency similar to that of the repetitive input are strengthened during stimulation. After stimulation, these synaptic changes persist and result in enhanced neural activity at the resonance frequency of these circuits. We have tested whether this explanation holds true in a neural network simulation (for simulation details see ‘Text S1). The results show that stimulation at a certain input frequency selectively strengthens those synapses that are incorporated in neural circuits that have a similar resonance frequency as the input, and weakens synapses incorporated in neural circuits with different resonance frequencies (cf. Fig. 2).

While tDCS effects neural tissue via a sustained modulation of the membrane voltage of neurons, tACS most probably yields its effect via an up- and down-regulation of certain synapses as indicated above. This lets us assume that tACS – like repetitive TMS [59] – should be better suited to modulate those cognitive functions that are closely related to brain oscillations at specific frequencies [62]. TDCS, on the other hand, can up- or down-regulate the stimulated brain areas irrespective of the frequency at which neurons in this area oscillate. Thus, tDCS might be more effective in regulating those brain functions that are clearly related to certain brain areas rather than certain EEG frequencies, e.g. syntax processing in Broca's area [63], face processing in the fusiform gyrus ([64], or motor processes in the primary motor cortex – to name but a few. It should be noted, however, that most probably no cognitive function is related to only one brain are or EEG oscillations (see, e.g. [65]). Nevertheless, modulating either of the two into the right direction may very well support a disturbed function.

In summary, our study revealed that tACS but not sham stimulation elevates EEG alpha power and thus demonstrates the feasibility of tACS to modulate specific oscillatory brain activity. Furthermore, the present findings strongly recommend tACS as a powerful tool for investigating human brain oscillations and indicate its feasibility for therapeutic applications.

## Supporting Information

Text S1 Details on the network simulation.
(0.04 MB DOC)
