# Supplementary material for: Transcranial Alternating Current Stimulation Enhances Individual Alpha Activity in Human EEG
Source: PLoS One. 2010 Nov 1;5(11):e13766. doi: 10.1371/journal.pone.0013766 (PMC2967471; doi:10.1371/journal.pone.0013766)
Supplement: Text S1 — Details on the network simulation. (0.04 MB DOC) [file pone.0013766.s001.doc]

**10-PONE-RA-19857 - Supporting information:**

*Network configuration*

We simulated a highly simplified network of spiking neurons consisting of a single exitatory driving neuron and a hidden layer of 2500 exitatory neurons. Each of the hidden layer neurons received a projection from and back to the driving neuron, resulting in 2500 recurrent loops. The total synaptic delay of each recurrent loop, e.g., the sum of the synaptic delays of projection and back-projection, was drawn randomly from a uniform distribution in the interval [20 160] ms. Synaptic weights were drawn randomly from a uniform distribution in the interval [0.1 0.6] for projections from the driving neuron to hidden layer neurons, and from a uniform distribution in the interval [0.00002 0.0008] for back-projections.

*Neuron Model*

Single neurons were simulated using the neuron model introduced by Izhikevich (2003) [1]. This model allows for fast computation, the configuration of different behaviors via a few parameters, and has already been used sucessfully in the simulation of EEG data [2]. It reduces the biophysically accurate Hodgkin-Huxley model to a two-dimensional system of ordinary differential equations

*dv/dt = 0.04v^2^ +5v +140−u + I,*

*du/dt = a(bv −u),*

where *t* denotes time, *v* and *u* are dimensionless variables, and *a*, *b*, *c*, and *d* are dimensionless real parameters. An auxiliary after-spike resetting occurs if *v* ≥ 30mV: v is reset to a value *c*, and *u* is incremented by a value *d*. The variable *v* can be interpreted as the membrane potential and *u* represents a membrane recovery variable that accounts for the activation of K^+^ ionic currents and inactivation of Na^+^ ionic currents. All inputs into the cell (synaptic *I_syn_* as well as external *I_ext_*) are collapsed into the dimensionless variable *I := I_syn_ + I_ext_*. The simulated time steps were 0.25 ms.

Model parameters of the excitatory neurons were chosen according to the simulations in Izhikevich et al., (2004) [3]: (a, b) = (0.02, 0.2) and (c, d)=(−65, 8) + (15,−6) r^2^,where r is randomly selected from the uniform distribution in the interval [0, 0.5] to obtain a behaviour between regular spiking (RS, r =0) and intrinsically bursting (IB, r =0.5). The square of r biases the distribution towards the RS cells.

*Synapse Model*

Synaptic short-term dynamics (depression and facilitation) were implemented utilizing the dynamic synapse model by Markram et al. (1998) [4]:

*dR/dt = (1−R)/D,*

*dw/dt = (U −w)/F,*

*F* and *D* are time-constants as reported in Markram et al. (1998), *U* describes the available neurotransmitter in a resting state, and the product *Rw* is the fractional amount of available neurotransmitter.

Each spike received by the postsynaptic neuron results in an update of *R* and *w*

*R ← R−Rw*

*w ← w +U(1−w),*

with *U*=0.5, *F*=1000 and *D*= 800. The synaptic current of a neuron is calculated in each time step by

*I_syn_ = g_AMPA_ ∙ v +g_NMDA_ [(v +80) /60]2/[1+[(v +80) /60]^2^]∙v*

where g_k_ denotes the time dependent synaptic conductance and *v* the actual membrane po- tential. The conductances change by first-order linear kinetics

*dg_k_/dt = −g_k_ ∙ τ_k_*

with time constants τ_k_ = 5 and 150 ms for the simulated AMPA and NMDA receptor types, respectively [3]. The rising time of currents is typically short and neglected.

If a spike is transmitted from presynaptic neuron i to postsynaptic neuron the conductances are updated depending on the synaptic efficiency *R_i_ · w_i_* and the synaptic weight *s_ij_*:

*g_k_ ←g_k_ + R_i_ · w_i_ · c_ij_,*

*Synaptic plasticity*

To enable learning in the model, we utilized the spike-timing dependent plasticity rule (STDP, [3,5] in which the temporal order of presynaptic and postsynaptic spike determines, whether a synapse is potentiated (LTP) or weakened (LTD):

A_+_∙exp(-|Δt|/τ_+_ ∙(1-s_ij_) if Δt > 0;

Δs_ij_ =

A-∙exp(-|Δt|/τ- ∙s_ij_ if Δt > 0;

with Δt denoting the difference between a postsysnaptic and the presynaptic spike, A_+_ = 0.000025, A_−_ = 0.000025, τ+ =15ms and τ− =20ms [6].

To avoid excessive increase or decrease of synaptic weights during STDP, we implemented a bidirectional synaptic scaling mechanism (cf. [7,8]). Turrigiano (2008) suggested that bidirectional synaptic scaling might recruit two different signaling pathways providing two independent scaling factors for upscaling and for downscaling of synaptic weights. We implemented this approach by assuming the scaling factor SF to be composed of two independent terms for downscaling and upscaling:

*SF = 1 + max(-0.95, min(0.95, 0.5 ∙ (exp(0.2 ∙ (f_min_-f))-exp(0.2∙(f-f_max_))))) ∙ 2∙10^-5^*

with *f_min_* and *f_max_* denoting the lower and upper limits for the firing rate *f* [9].

Assuming *f_min_*= 0 Hz and *f_max_*= 80, synapses are scaled up if postsynaptic neurons’ firing rate is below 10 Hz, and scaled down if the firing rate is above 50 Hz.

The driving neuron was stimulated by setting the external input *I_ext_* to 16 for 5 ms every 100 ms, resulting in a 10 Hz stimulation frequency.

**References:**

1. Izhikevich E.M. (2003). Simple model of spiking neurons**.** IEEE Trans. Neural Netw. *14***:** 1569-1572.

2. Frund I., Ohl F.W., and Herrmann C.S. (2009). Spike-timing-dependent plasticity leads to gamma band responses in a neural network**.** Biol. Cybern. 227-240.

3. Izhikevich E.M., Gally J.A., and Edelman G.M. (2004). Spike-timing dynamics of neuronal groups**.** Cereb. Cortex *14***:** 933-944.

4. Markram H., Wang Y., and Tsodyks M. (1998). Differential signaling via the same axon of neocortical pyramidal neurons**.** Proc. Natl. Acad. Sci. U. S. A *95***:** 5323-5328.

5. Bi G.Q. and Poo M.M. (1998). Synaptic modifications in cultured hippocampal neurons: dependence on spike timing, synaptic strength, and postsynaptic cell type**.** J. Neurosci. *18***:** 10464-10472.

6. Handrich, S., Herzog, A., Wolf, A., & Herrmann, C. S. (2009). A Biologically PlausibleWinner-Takes-All Architecture. In D. Huang, K. Jo, H. Lee, H. Kang, & V. Bevilacqua, Emerging Intelligent Computing Technology and Applications. With Aspects of Artificial Intelligence, Lecture Notes in Computer Science (Vol. 5755, pp. 315-326). Berlin, Heidelberg: Springer Berlin Heidelberg. doi: 10.1007/978-3-642-04020-7_34.

7. Abbott L.F. and Nelson S.B. (2000). Synaptic plasticity: taming the beast**.** Nat. Neurosci. *3 Suppl***:** 1178-1183.

8. Turrigiano G.G. (2008). The self-tuning neuron: synaptic scaling of excitatory synapses**.** Cell *135***:** 422-435.

9. Rach S, Herzog A, Herrmann CS (2010). Bidirectional synaptic scaling is necessary for implementing reinforcement learning via spike-timing-dependent-plasticity. Front. Comput. Neurosci. Conference Abstract: Bernstein Conference on Computational Neuroscience. doi: 10.3389/conf.fncom.2010.51.00081
